# Emergence of Dengue Virus Serotype 2 Cosmopolitan Genotype, Colombia

**DOI:** 10.3201/eid3001.230972

**Published:** 2024-01

**Authors:** David Martínez, Marcela Gómez, Carolina Hernández, Marina Muñoz, Sandra Campo-Palacio, Marina González-Robayo, Marcela Montilla, Norma Pavas-Escobar, Juan David Ramírez

**Affiliations:** Universidad del Rosario, Bogotá, Colombia (D. Martínez, M. Gómez, C. Hernández, M. Muñoz, J.D. Ramírez);; Universidad de Boyacá, Tunja, Colombia (M. Gómez);; Centro de Tecnología en Salud (CETESA), Innovaseq SAS, Bogotá (C. Hernández);; Laboratorio de Salud Pública, Secretaría de Salud Departamental Meta, Villavicencio, Colombia (S. Campo-Palacio, M. González-Robayo, M. Montilla, N. Pavas-Escobar);; Universidad Cooperativa de Colombia, Villavicencio, Colombia (M. Montilla, N. Pavas-Escobar);; Icahn School of Medicine at Mount Sinai, New York, New York, USA (J.D. Ramírez)

**Keywords:** dengue virus, viruses, vector-borne infections, DENV-2, transmission, Colombia, cosmopolitan genotype

## Abstract

Using Oxford Nanopore technologies and phylogenetic analyses, we sequenced and identified the cosmopolitan genotype of dengue virus serotype 2 isolated from 2 patients in the city of Villavicencio, Meta department, Colombia. This identification suggests the emergence of this genotype in the country, which warrants further surveillance to identify its epidemic potential.

Dengue fever is a viral disease transmitted by *Aedes* spp. mosquitoes; the Americas are one of the most severely affected regions (*1*). The causative agent of dengue fever is the dengue virus (DENV), a positive-sense single-stranded RNA virus with a genome size of ≈10.7 kilobase. This virus is categorized into 4 distinct serotypes (DENV-1–4), classified on the basis of their surface antigens, and each serotype further consists of different genotypes that are phylogenetically distinct (*2*,*3*).

Recent epidemics in South America have been primarily attributed to the DENV-2 serotype, according to epidemiologic reports from the region (*4*). In Colombia, 70,418 cases of dengue fever have been reported as of August 2023; DENV-2 has been identified in most cases (*5*). Currently, this serotype consists of 5 genotypes named according to the region in which they circulate. Asian I and II genotypes are predominantly found in Asia, whereas the American genotype, which is no longer in circulation, was once prevalent in Central and South America. In the 1980s, the American genotype was replaced by the Asian-American genotype, which now circulates in Southeast Asia and the Americas. Last, the cosmopolitan genotype is noteworthy for its extensive global distribution, spanning 5 continents (*6*).

The cosmopolitan genotype has recently expanded in Africa and the Americas (*7*). This widespread dispersal has led to substantial intragenotype heterogeneity, reflecting the evolutionary forces acting within this genotype that are associated with its transmission. An outbreak attributed to the cosmopolitan genotype was reported in Madre de Dios Province, Peru, in 2019, coinciding with its recent expansion in Africa (*8*,*9*). In 2021, an additional 2 reports were documented in the states of Acre and Goiás in Brazil (*4*). Those reports shed light on a potential introduction route of the genotype into Brazil, specifically from the border with Peru (*4*). In 2023, the World Health Organization reported an outbreak in Latin America, generating a state of alert because of the increase in DENV cases (*10*). The genetic characteristics acquired during the extensive dissemination of the cosmopolitan genotype emphasize the need for further research into its diversity, evolution, and transmission dynamics within DENV-endemic areas.

In this report, we discuss 2 cases of the cosmopolitan genotype DENV-2 identified in Villavicencio, a city in the Meta department of Colombia. Of note, this department had the highest number of DENV cases in Colombia in 2023, accounting for 15.4% (10,859 cases) of total cases reported nationwide as of August (*5*). The 2 cases involved 2 young men with no travel history residing in suburban neighborhoods in southern Villavicencio (Figure, panel A). Both patients exhibited symptoms of fever, headache, myalgia, intense and continuous abdominal pain, and a platelet count of <100,000. Those symptoms align with the classification of DENV infection with warning signs, and dates of symptom onset were April 26, 2023, and April 29, 2023.

**Figure:**
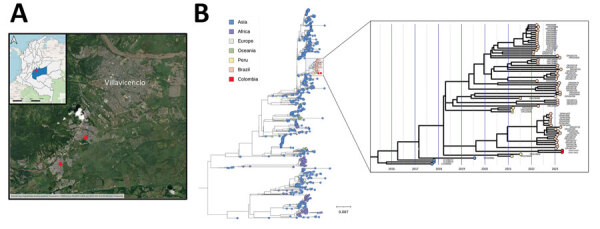
Phylogenetic analysis of dengue virus 2 cosmopolitan genotype, Colombia. A) Geographic location of the neighborhoods where the patients’ residences are situated. B) Maximum-likelihood tree rooted at the midpoint depicts the evolutionary relationships of the complete genome sequence of the dengue virus 2 cosmopolitan genotype identified in 2 patients from the city of Villavicencio in Meta department, Colombia (red circles), along with 1,001 publicly available sequences from GenBank. The highlighted blue area is shown in a time-resolved maximum-likelihood tree in expanded panel; colors represent different sampling locations. Scale bar indicates number of substitutions per site.

Serum samples were collected and sent to the microbiology laboratory at Universidad del Rosario in Bogotá, Colombia for processing. We extracted viral RNA using the Quick-RNA Viral Kit (Zymo Research, https://zymoresearch.eu). The infection was confirmed to be caused by the DENV-2 serotype using the previously described protocol (Appendix). We performed whole-genome sequencing using MinION (Oxford Nanopore Technology, https://nanoporetech.com) to determine the corresponding genotype classification and to conduct subsequent analysis of the local distribution of DENV (Appendix). The Technical Research Committee and Ethics Research Board from Universidad del Rosario in Bogotá, Colombia approved the protocol implemented in this study (approval no. DVO005 1585-CV142).

We conducted an initial maximum-likelihood phylogenetic analysis to identify the genotype. The analysis revealed that the sequences obtained from the patients were closely related, belonged to the DENV-2 cosmopolitan genotype, and were placed within the South America sequences found in Tefé and Tabatinga, Brazil, and Madre de Dios in Peru (Figure, panel B).

Further examination using a time-resolved maximum-likelihood tree demonstrated that those sequences were closely related to sequences reported in the Tabatinga province in Brazil. The bootstrap support for this relationship was 95% (Figure, panel B). This finding suggests potential cross-border transmission in the Tabatinga province, highlighting the possibility of viral spread across borders.

In conclusion, although genetic data alone cannot provide conclusive evidence about the directionality of the introduction of the DENV-2 cosmopolitan genotype, insights gained from phylogenetic reconstruction and temporal information suggest a potential introduction from Tabatinga, Brazil, with subsequent spread northwards in Colombia. Tabatinga is located in the tripartite border region between Brazil, Colombia, and Peru adjacent to the Amazonas department in southern Colombia. Because of the limited research available on the cosmopolitan genotype, our understanding of its effects on dengue disease dynamics in Colombia remains incomplete. Further investigations are required to gain a more comprehensive insight into its potential for local, regional, and global epidemics. Our findings highlight the importance of implementing robust genomic surveillance in the region, especially considering the ongoing outbreak in Latin America.

Appendix Additional information for emergence of dengue virus serotype cosmopolitan genotype, Colombia.
