# Supplementary material for: Emergence of Dengue Virus Serotype 2 Cosmopolitan Genotype, Colombia
Source: Emerg Infect Dis. 2024 Jan;30(1):189–92. doi: 10.3201/eid3001.230972 (PMC10756373; doi:10.3201/eid3001.230972)
Supplement: Appendix — Additional information for emergence of dengue virus serotype cosmopolitan genotype, Colombia. [file 23-0972-Techapp-s1.pdf]

Article DOI: https://doi.org/10.3201/eid3001.230972

EID cannot ensure accessibility for supplementary materials supplied by authors. Readers who have difficulty accessing supplementary content should contact the authors for assistance.

Emergence of Dengue Virus Serotype 2 Cosmopolitan Genotype, Colombia

Appendix

Materials and Methods

Ethics Statement

The Technical Research Committee and Ethics Research Board from Universidad del Rosario in Bogotá, Colombia approved the protocol implemented in this study DVO005 1585-CV142: "Vigilancia molecular de Enfermedades transmitidas por vectores ETVs y enfermedades emergentes en la región de la Orinoquía."

Sample Collection, Viral RNA Isolation, and PCR identification

Serum samples were collected of patients from the department of Meta, Colombia and sent to the microbiology laboratory at Universidad del Rosario, Bogotá-Colombia for processing. Viral RNA was extracted using the Quick-RNA Viral Kit (ZYMO RESEARCH, https://zymoresearch.eu), and a traditional PCR (Polymerase Chain Reaction) test was performed for identification of dengue virus (DENV) serotypes using the previously described protocol (*1*), confirming the infection with DENV-2 serotype.

Library Preparation and Whole-Genome Sequencing

Retro transcription was performed from the genetic material to generate cDNA (complementary DNA) using the LunaScript RT SuperMix Reverse transcription Kit enzyme (NEB #E3010). Then, cDNA was used to conduct a multiplex PCR using DENV-2 sequencing primers (*2*) was conducted by using Q5 High Fidelity Hot-Start DNA Polymerase (New England Biolabs, https://www.neb.com) . The amplified products were sequenced by ONT (Oxford Nanopore Technology), for which barcode ligation (assigning one barcode per sample) was

initially performed using the ONT Barcode Kit (EXP-NBD196). Subsequently, the library was constructed by joining the amplicons in equal volumes (with their barcode ligated) and proceeded to adaptor ligation using the ONT ligation sequencing kit (SQK.LSK109). The constructed library was sequenced in ONT MinION using R.9.4 flow cells and MinKnow V.3.1.4 software, getting ≈500,000 reads per sample. The bioinformatics analysis was performed on the raw Fast5 files, using Super Accuracy base-calling (SUP) (Q>10) to obtain the Fastq files and then demultiplexed with the Guppy V3.1.5 tool (*3*).

Generation of Consensus Sequences and Phylogenetic Analysis

The generated reads were initially mapped against the DENV-2 reference genome (NC_001474) available in the National Center for Biotechnology Information (NCBI) database to obtain consensus sequences using Minimap2 V.2.24 (*4*). Subsequently, an assembly correction was performed using the Fast5 files and Fastq files obtained from the assembly, using Nanopolish V.0.8.4 tool. A phylogenetic reconstruction was generated on the two sequences obtained previously and reference sequences obtained from GenBank (Table). Sequence alignment was initially carried out using MAFFT V.5 (*5*). With this alignment, a Maximum Likelihood (ML) tree was constructed in iQtree tool version 1.6.12 (*6*) using the substitution model GTR+F+G4 (Model chosen according to the BIC generated by ModelFinder included in the IQ tree tool version 1.6.12). Node support was performed using 1,000 iterations by the Ultrafast Bootstrap (UFBoot) approach and visualized in iTOL We inferred time-scaled trees by using TreeTime (*7*) which considers a fixed clock rate of 8x10−3, a strict clock (SC) under a coalescent tree skyline prior and a root step to minimize residuals in a root-to-tip.

References

1. Chien LJ, Liao TL, Shu PY, Huang JH, Gubler DJ, Chang GJJ. Development of real-time reverse transcriptase PCR assays to detect and serotype dengue viruses. J Clin Microbiol. 2006;44:1295–304. **PMID 16597854**

2. Stubbs SCB, Blacklaws BA, Yohan B, Yudhaputri FA, Hayati RF, Schwem B, et al. Assessment of a multiplex PCR and Nanopore-based method for dengue virus sequencing in Indonesia. Virol J. 2020;17:24. **PMID 32054488**

3. Wick RR, Judd LM, Holt KE. Performance of neural network basecalling tools for Oxford Nanopore sequencing. Genome Biol. 2019;20:129. **PMID 31234903**

4. Li H. Minimap2: pairwise alignment for nucleotide sequences. Bioinformatics. 2018;34:3094–100. **PMID 29750242**

5. Katoh K, Kuma K, Toh H, Miyata T. MAFFT version 5: improvement in accuracy of multiple sequence alignment. Nucleic Acids Res. 2005;33:511–8. PubMed https://doi.org/10.1093/nar/gki198

6. Nguyen LT, Schmidt HA, von Haeseler A, Minh BQ. IQ-TREE: a fast and effective stochastic algorithm for estimating maximum-likelihood phylogenies. Mol Biol Evol. 2015;32:268–74. **PMID 25371430**

7. Sagulenko P, Puller V, Neher RA. TreeTime: Maximum-likelihood phylodynamic analysis. Virus Evol. 2018;4:vex042. **PMID 29340210**

Appendix Table 1. Sequencing statistics of the genomes obtained in this research.

Acc Number	Number of Reads	Genome Coverage	Coverage Depth*	Mean read length	Mean read quality
OR619404	9,451	92.2%	120X	137	13.9
OR619405	3,843	92%	47X	133	13.7

*Depth coverage was calculated with the following formula: (Number of Reads * Mean read length)/(Reference genome length). Reference genome length: 10,723.

Appendix Table 2. List of accession numbers, locations, collection date for dengue virus serotype 2 cosmopolitan genotypes obtained from GenBank.

Accession no.	Location	Collection date	Accession	Location	Collection date
MW481671	Angola	2018–7-9	KR779782	Singapore	2007–4-8
MZ130520	Democratic Republic of the Congo	2016–4-11	MW512342	Singapore	2007–1-10
MZ130521	Democratic Republic of the Congo	2016–6-20	JN851120	Singapore	2007–12–29
MW191701	India	2017–7-1	MW512350	Singapore	2008–1-8
MW191700	India	2017–7-1	JN851122	Singapore	2008–7-29
ON231305	Senegal	2018–10–29	MW512347	Singapore	2007–1-1
MZ130526	Tanzania	2014–5-21	MW512348	Singapore	2007–9-13
MZ130522	Egypt	2017–11–7	MW946584	Thailand	2007–11–15
MZ130528	Djibouti	2018–6-24	KY586700	Thailand	2009–12–31
MZ130527	France	2019–1-5	JN851119	Singapore	2008–4-8
MZ130519	Mozambique	2019–3-5	GQ398265	Singapore	2008–1-1
MZ130518	France	2019–5-14	GQ398266	Singapore	2007–12–31
MZ130524	Benin	2019–7-9	MW512354	Singapore	2009–1-1
MZ130525	Cameroon	2019–12–29	MW512355	Singapore	2009–11–24
OR025669	Brazil	2023–3-27	KM279609	Singapore	2009–1-29
OR025670	Brazil	2023–3-27	KM279610	Singapore	2010–5-31
MW946478	India	1974–1-1	KM279596	Singapore	2012–10–26
FJ882602	Sri Lanka	1996–1-1	KM279518	Singapore	2011–6-15
MG560143	India	2014–1-1	KM279528	Singapore	2012–1-1
MG721056	India	2016–1-1	KM279519	Singapore	2011–8-20
MW512470	Singapore	2017–12–31	KM279566	Singapore	2012–4-4
OM639983	India	2021–11–16	KM279577	Singapore	2012–9-11
OM639986	India	2021–11–16	KM279580	Singapore	2013–1-1
OM648092	India	2021–10–20	KX380823	Singapore	2012–12–31
OM681318	India	2021–11–16	KM279573	Singapore	2012–10–7
KY427084	India	2010–9-1	KX380825	Singapore	2013–1-1
MH822949	India	2014–10–28	KX380837	Singapore	2013–3-17
MK473384	Kenya	2016–5-4	KR779786	Singapore	2013–12–31
MK473386	Kenya	2016–5-4	KX380816	Singapore	2012–12–31
MG779195	Kenya	2017–1-1	KX380827	Singapore	2013–1-1
MK473385	Kenya	2016–5-10	KM279522	Singapore	2011–5-20

Accession no.	Location	Collection date	Accession	Location	Collection date
MN577554	Kenya	2017–6-30	KM279513	Singapore	2011–7-21
MN335246	Kenya	2017–6-7	KM279514	Singapore	2011–7-21
MN335244	Kenya	2017–5-29	KM279581	Singapore	2011–7-1
MN335247	Kenya	2017–4-10	KM279523	Singapore	2011–8-18
MG779196	Kenya	2017–1-7	KM279526	Singapore	2011–7-1
MG779201	Kenya	2017–1-1	KM279563	Singapore	2012–6-20
MG779197	Kenya	2017–3-18	MW512383	Singapore	2013–7-3
MG779203	Kenya	2017–1-11	MW512382	Singapore	2013–2-5
MG779198	Kenya	2017–1-1	KX380831	Singapore	2013–4-10
MN577556	Kenya	2017–7-31	KM279539	Singapore	2012–5-12
MN577553	Kenya	2017–6-30	KM279537	Singapore	2012–7-9
MN577555	Kenya	2017–7-1	KM279587	Singapore	2012–6-9
MZ857209	Kenya	2013–1-1	KM279592	Singapore	2012–12–9
MH456899	Kenya	2014–6-11	KM279549	Singapore	2012–10–4
MH456898	Kenya	2014–6-23	KM279591	Singapore	2012–10–4
MH456897	Kenya	2014–7-9	MK578532	China	2016–4-1
MH456892	Kenya	2014–10–17	MW512448	Singapore	2015–1-12
MH456893	Kenya	2014–11–25	MW512466	Singapore	2016–1-1
MH456894	Kenya	2014–6-20	OQ028230	Vietnam	2020–9-7
MH456895	Kenya	2014–7-4	MN982899	Australia	2019–5-1
MN577559	Kenya	2013–4-1	OQ028232	Vietnam	2020–3-2
MN577560	Kenya	2013–5-16	OQ832621	Vietnam	2019–12–1
MN577561	Kenya	2013–5-16	OQ832624	Vietnam	2020–9-1
MN577557	Kenya	2017–7-1	OQ832625	Vietnam	2020–9-30
MN577552	Kenya	2014–2-28	KU666946	Malaysia	2014–2-28
MN577551	Kenya	2013–4-30	MW512430	Singapore	2014–12–31
MN577563	Kenya	2013–5-31	KX380830	Singapore	2013–10–25
MN577565	Kenya	2014–1-31	KX380835	Singapore	2013–1-8
MN577558	Kenya	2017–7-31	MW512406	Singapore	2013–12–31
LC367234		2009–10–8	MW512407	Singapore	2013–1-1
MW512372	Singapore	2012–8-9	KX380809	Singapore	2012–1-1
OP310806	India	2018–10–30	KX380822	Singapore	2012–12–31
MH891770	India	2018–4-23	KX380832	Singapore	2013–1-1
MK858109	India	2016–9-11	KX380810	Singapore	2012–12–31
MK858116	India	2017–9-6	KX380838	Singapore	2013–1-1
ON123649	India	2020–9-23	MW512404	Singapore	2013–12–17
OM639989	India	2021–11–16	MN952966	China	2015–11–1
OM639987	India	2021–11–16	MW512464	Singapore	2016–1-12
OM639988	India	2021–11–16	MH827525	China	2017–1-1
MK858102	India	2016–10–13	MN018357	China	2014–5-28
OP310807	India	2019–9-26	MN923112	China	2019–8-26
MG721054	India	2016–1-1	OM349569	China	2019–9-25
MK858100	India	2016–8-10	OM368351	China	2019–9-16
MK858104	India	2016–10–5	ON890422	China	2019–9-16
MG592698	India	2016–12–31	ON908229	China	2019–9-16
MK858099	India	2016–8-26	MH827539	China	2017–1-1
MK858106	India	2016–10–20	MN018353	China	2016–6-15
JX475906	India	2009–1-1	MW512465	Singapore	2016–6-12
MG560144	India	2014–12–31	MW730832	Nepal	2017–7-1
LC121816		2016–1-31	MW730816	Nepal	2017–7-22
MH822950	India	2013–8-19	MW730815	Nepal	2017–7-1
MH822951	India	2013–9-4	MW730822	Nepal	2017–7-1
MH822952	India	2014–9-16	MW730826	Nepal	2017–7-31
MW512449	Singapore	2016–12–31	MW730828	Nepal	2017–7-31
LC436670	Bangladesh	2017–10–1	MW730829	Nepal	2017–7-31
LC436671	Bangladesh	2017–10–1	JN851123	Singapore	2004–1-1
MW730836	Nepal	2017–7-31	JN851126	Singapore	2005–12–31
MW730837	Nepal	2017–7-1	HM488257	Guam	2001–4-19
MW730838	Nepal	2017–7-31	JF327392	Singapore	2009–1-1
MH822953	India	2015–9-9	KC762669	Indonesia	2007–7-17
MH822956	India	2013–9-9	MW512368	Singapore	2011–1-1
MH822955	India	2014–10–25	KU509268	Indonesia	2009–12–31
MK858096	India	2014–11–10	MW512366	Singapore	2011–12–31
MH822954	India	2015–8-31	MW512402	Singapore	2013–1-1
MK629885	South Korea	2015–11–1	MH048672	Malaysia	2014–12–1
MW481692	Angola	2018–6-1	KC131142	China	2012–9-1
MW481676	Angola	2018–4-4	MW512401	Singapore	2013–12–31
MW481677	Angola	2018–3-31	MN018356	China	2014–9-23
MW481679	Angola	2018–4-7	MW721472	China	2014–10–1

Accession no.	Location	Collection date	Accession	Location	Collection date
MW481680	Angola	2018–5-7	MW512447	Singapore	2015–12–31
MW481674	Angola	2018–4-16	MW512463	Singapore	2016–1-1
MW481673	Angola	2018–4-22	MK411558	Indonesia	2016–2-20
MK564488	China	2018–3-9	MK411559	Indonesia	2016–2-23
MW481682	Angola	2018–4-24	OP984834	Vietnam	2022–11–20
MW481686	Angola	2018–4-17	MN923116	China	2019–6-30
MK578533	China	2018–8-16	OR125606	China	2023–5-6
MK783207	China	2018–4-23	OR136164	Indonesia	2023–5-9
MK783206	China	2018–8-19	MW512416	Singapore	2014–1-1
MK783210	China	2018–12–31	MW512452	Singapore	2016–1-1
MK783209	China	2018–12–31	MW512435	Singapore	2015–1-1
MW481685	Angola	2018–4-7	MN018337	China	2015–6-20
MW481691	Angola	2018–5-13	KX621246	China	2015–2-4
MW481688	Angola	2018–4-17	KX621247	China	2015–1-1
MW481687	Angola	2018–5-14	MK783190	China	2018–12–31
MW481694	Angola	2018–5-17	MW721460	China	2018–10–1
MH460898	Angola	2018–1-18	MK564479	China	2016–8-23
MW481675	Angola	2018–3-12	MH827540	China	2017–1-1
MW481689	Angola	2018–5-15	MN018340	China	2016–7-7
MW481693	Angola	2018–5-20	MN018348	China	2016–7-18
MW481684	Angola	2018–3-31	MH827543	China	2017–1-1
MW481670	Angola	2019–2-15	MN018347	China	2016–10–10
MW481690	Angola	2018–3-31	MW721469	China	2017–10–1
JQ922551	India	2005–12–31	MW721471	China	2014–10–31
AF359579		1999–1-1	MK783201	China	2018–12–17
KF041236	Pakistan	2008–1-1	MW721461	China	2018–10–1
JQ955623	India	2009–1-1	OQ426773	Vietnam	2018–10–22
JQ922549	India	1996–11–13	OQ426757	Vietnam	2018–10–15
MZ857219	Somalia	1996–12–31	OM730078	India	2021–11–16
GQ252676	Sri Lanka	2003–8-28	OP921001	India	2022–7-27
GQ252677	Sri Lanka	2004–1-1	OP921000	India	2022–7-29
KF360005	Pakistan	2010–11–1	OM680963	India	2021–11–16
KF041233	Pakistan	2011–1-1	OM639993	India	2021–11–16
KF041237	Pakistan	2009–1-1	OM639980	India	2021–11–16
KJ830750	Saudi Arabia	2014–1-13	OM700180	India	2021–11–16
KF041235	Pakistan	2009–1-1	OQ821481	India	2022–7-1
KJ010186	Pakistan	2013–10–2	OQ821482	India	2022–8-19
KF041232	Pakistan	2011–1-16	OM639981	India	2021–9-2
KF041234	Pakistan	2011–1-16	OM639982	India	2021–9-14
KJ010185	Pakistan	2011–10–20	OM639992	India	2021–11–16
KM217156	Pakistan	2011–8-27	OM639984	India	2021–11–16
KM217157	Pakistan	2011–8-27	OP809582	India	2021–12–31
KM217158	Pakistan	2013–8-23	OP389112	China	2022–7-25
KJ701507	Pakistan	2013–8-27	MW512451	Singapore	2016–1-1
DQ448231	India	2001–1-1	LC410189	Thailand	2016–10–1
FJ898454	India	2006–1-1	LC410190	Thailand	2016–10–1
KU509271	India	2006–12–18	LC410191	Thailand	2017–2-1
JQ955624	India	2011–6-7	MK783192	China	2018–6-8
OP310808	India	2019–9-27	MK564484	China	2018–7-23
ON123652	India	2020–2-28	MK783191	China	2018–12–31
OP809581	India	2021–1-1	MK783199	China	2018–6-5
OM639991	India	2021–11–16	MK783196	China	2018–1-1
OM639990	India	2021–11–16	MK783197	China	2018–7-12
OM698821	India	2021–11–16	MK783198	China	2018–12–31
MZ857222	Djibouti	2007–12–30	MK783194	China	2018–2-4
MZ857218	Eritrea	2017–1-8	MK783193	China	2018–4-1
MZ857221	Uganda	2017–1-8	MK783195	China	2018–4-1
MH822939	India	2012–11–8	OP684209	China	2019–10–12
MW512412	Singapore	2014–1-1	OP684199	China	2019–7-31
MH891768	India	2016–10–26	OP684210	China	2019–10–15
MH822940	India	2014–12–31	MT754369	China	2019–10–24
MH822941	India	2013–8-30	MT754367	China	2019–10–24
KY427085	India	2011–1-3	MZ453008	China	2019–12–31
MN294937	Saudi Arabia	2016–2-2	MZ453010	China	2019–1-1
KF479233	China	2013–1-4	MZ453009	China	2019–8-31
MH822944	India	2012–10–8	MZ453011	China	2019–12–31
MW512413	Singapore	2014–2-22	MZ636804	Thailand	2019–11–30
MH822943	India	2014–11–7	OR029723	China	2019–8-17
MH822946	India	2012–10–19	OR029721	China	2019–8-16

Accession no.	Location	Collection date	Accession	Location	Collection date
MH822945	India	2012–10–19	OR029732	China	2019–8-21
MK858097	India	2013–1-1	OR029722	China	2019–8-17
MH822947	India	2012–9-24	OR029728	China	2019–8-20
MH822948	India	2012–11–20	OR029730	China	2019–8-20
MW191698	India	2016–7-1	ON888666	China	2019–8-16
KX380829	Singapore	2013–12–31	OR029720	China	2019–8-16
MW191699	India	2016–7-31	MW720952	China	2019–9-1
MN253134	India	2017–10–30	MW512387	Singapore	2013–1-1
MK858105	India	2016–10–5	LC436669	Bangladesh	2017–10–1
MK858112	India	2017–10–5	LC436674	Bangladesh	2017–11–1
OM700181	India	2021–11–16	LC436673	Bangladesh	2017–11–1
OM639985	India	2021–11–16	MN328061	Bangladesh	2019–7-29
ON123663	India	2018–9-17	ON123639	Peru	2021–3-4
ON109598	India	2021–1-1	ON123644	Peru	2021–7-21
ON799267	India	2018–12–28	ON123640	Peru	2021–3-8
MG721057	India	2016–1-1	ON123638	Peru	2021–3-1
MW512468	Singapore	2017–12–31	ON123641	Peru	2021–3-24
MG721058	India	2016–12–31	OR150734	Brazil	2023–1-24
MK858111	India	2016–9-8	OR150740	Brazil	2023–2-13
MG721055	India	2016–1-26	OR150737	Brazil	2023–2-7
MK858107	India	2016–10–19	OR150733	Brazil	2023–1-29
MN272405	Seychelles	2016–5-7	OR150736	Brazil	2023–2-7
MN272404	France	2018–3-31	OR150741	Brazil	2023–1-13
MK858101	India	2016–8-10	OR150732	Brazil	2023–1-27
MK858103	India	2016–10–13	OR150712	Brazil	2023–4-5
MG721062	India	2016–11–26	OR150709	Brazil	2023–1-17
MK858110	India	2016–9-8	OR150705	Brazil	2023–2-14
MK858098	India	2016–8-31	OR150711	Brazil	2023–3-27
MK858108	India	2016–10–5	OR150706	Brazil	2023–2-26
OQ339137	India	2022–1-1	OP941844	Brazil	2022–11–6
OQ733364	India	2022–1-1	OP941839	Brazil	2022–10–24
OQ733363	India	2022–1-22	OP941840	Brazil	2022–10–24
OQ547234	India	2022–11–7	OP941842	Brazil	2022–10–18
ON123651	India	2020–10–22	OP941836	Brazil	2022–11–1
OP310805	India	2020–10–14	OP941838	Brazil	2022–10–19
OQ733365	India	2022–1-1	OP941837	Brazil	2022–11–3
MH822942	India	2014–9-22	OR138993	Brazil	2022–4-21
MW387614	China	2009–1-31	OR150727	Brazil	2023–2-19
MN018339	China	2014–6-20	OR150718	Brazil	2023–2-18
MN720746	France	2014–8-25	OR150721	Brazil	2023–2-16
MW512469	Singapore	2017–10–10	OR138998	Brazil	2021–7-14
OP410991	Singapore	2017–10–1	OR150708	Brazil	2023–1-17
MN018358	China	2016–2-1	OR150739	Brazil	2023–2-13
MK629886	South Korea	2015–1-1	OR150735	Brazil	2023–2-5
MK578531	China	2016–2-4	OR150738	Brazil	2023–2-10
MH110583	China	2017–9-1	OR025672	Brazil	2023–3-5
OP684160	China	2017–8-30	OR025675	Brazil	2023–3-16
OP684169	China	2017–9-3	OR025600	Brazil	2023–3-23
MH110589	China	2017–9-1	ON634750	Brazil	2022–3-24
MH110585	China	2017–9-1	ON634756	Brazil	2022–1-30
OP684161	China	2017–8-31	ON634742	Brazil	2022–2-5
MH110575	China	2017–8-1	OR150729	Brazil	2023–2-13
OP684154	China	2017–8-28	OR150730	Brazil	2023–2-13
MF156247	China	2015–10–28	OR039513	Brazil	2022–4-1
MF156248	China	2015–10–28	OR150726	Brazil	2023–2-2
MF156236	China	2015–10–7	OR150717	Brazil	2023–1-27
MF156237	China	2015–10–18	OR150722	Brazil	2023–2-19
MF156238	China	2015–10–18	OR150725	Brazil	2023–1-25
MF156243	China	2015–10–28	OR150723	Brazil	2023–2-10
MF156245	China	2015–10–28	OR150720	Brazil	2023–4-10
MF156246	China	2015–10–28	OR150719	Brazil	2023–2-17
MF156242	China	2015–10–26	OR138984	Brazil	2022–4-15
MF156244	China	2015–10–28	OR138989	Brazil	2022–4-12
KY937186	China	2015–12–31	OR138990	Brazil	2022–4-19
KY937188	China	2015–12–31	OR138992	Brazil	2022–4-13
KY672951	China	2015–11–13	OR039516	Brazil	2022–4-18
KY672953	China	2015–7-20	OR039505	Brazil	2022–4-1
MF940251	China	2015–1-1	OR039502	Brazil	2022–4-1
MF940252	China	2015–3-20	OR039500	Brazil	2022–3-28

Accession no.	Location	Collection date	Accession	Location	Collection date
MF940236	China	2015–2-14	OR039507	Brazil	2022–4-13
KY672950	China	2015–9-28	OR039496	Brazil	2022–3-24
MF940239	China	2015–2-13	OR039497	Brazil	2022–3-28
MF940242	China	2015–1-1	OR039508	Brazil	2022–4-22
MF940246	China	2015–2-12	OR039499	Brazil	2022–3-30
MF940237	China	2015–7-7	OR039501	Brazil	2022–4-1
MF940243	China	2015–6-22	OR039503	Brazil	2022–4-2
MF940241	China	2015–10–18	OR039504	Brazil	2022–4-2
MF940238	China	2015–10–30	KX452021	Malaysia	2014–1-1
MF940253	China	2015–12–31	KX452022	Malaysia	2014–1-1
GQ398258	Indonesia	1975–1-1	KX452034	Malaysia	2014–1-31
GQ398259	Indonesia	1976–5-7	MH488959	Malaysia	2014–1-1
GQ398262	Indonesia	1976–1-1	KX452040	Malaysia	2014–1-31
GQ398260	Indonesia	1976–3-31	KX452041	Malaysia	2014–1-1
GQ398261	Indonesia	1976–12–11	MH827536	China	2017–1-1
MN577562	Kenya	2013–5-31	MN018346	China	2016–2-11
MN577564	Kenya	2014–6-1	KU666947	Malaysia	2014–2-1
EU056810	Burkina Faso	1983–1-1	KX452026	Malaysia	2014–1-1
GU131843	Burkina Faso	1986–12–31	KU517847	Philippines	2015–8-3
OM317565	Cameroon	2020–6-24	KX372564	Australia	2015–3-22
OM317566	Cameroon	2020–8-2	MW512390	Singapore	2013–1-1
MT982126	Burkina Faso	2019–8-10	KX452032	Malaysia	2014–1-31
MT982148	Burkina Faso	2019–9-8	KX452017	Malaysia	2014–1-31
KY627762	Burkina Faso	2016–11–5	MH110573	China	2017–8-1
KY627763	Burkina Faso	2016–11–21	OP684152	China	2017–7-2
MT261965	Burkina Faso	2017–10–23	MW512398	Singapore	2013–1-1
MT261966	Burkina Faso	2017–11–8	KU666944	Malaysia	2014–1-1
OL469513	Cote D Ivoire	2017–5-4	MW512453	Singapore	2016–1-1
MT261958	Burkina Faso	2017–10–16	MW512394	Singapore	2013–1-1
MT261963	Burkina Faso	2017–10–25	KX452024	Malaysia	2014–1-1
MT261957	Burkina Faso	2017–10–16	KX452027	Malaysia	2014–1-31
MT261959	Burkina Faso	2017–10–17	KX452045	Malaysia	2014–1-1
LC666719	Ghana	2017–2-20	KX452023	Malaysia	2014–1-31
LC666718	Ghana	2017–9-2	MW512436	Singapore	2015–1-1
MW288030	Senegal	2018–10–29	MW512420	Singapore	2014–4-22
MW288029	Senegal	2018–10–1	MW512422	Singapore	2014–1-1
MW288024	Senegal	2018–10–1	MW512426	Singapore	2014–10–27
ON231312	Senegal	2018–11–8	MW512437	Singapore	2015–1-1
ON231311	Senegal	2018–11–9	MW512455	Singapore	2016–12–31
ON231313	Senegal	2018–11–9	MW512424	Singapore	2014–7-30
MT982169	Cote D Ivoire	2017–6-6	MK629884	South Korea	2015–9-19
MT261964	Burkina Faso	2017–10–24	KY921905	Singapore	2015–3-1
MT261969	Burkina Faso	2017–10–30	MK513444	Singapore	2015–1-30
MT261961	Burkina Faso	2017–10–26	MW512438	Singapore	2015–3-13
MT261971	Burkina Faso	2017–11–6	MW512446	Singapore	2015–6-14
ON885286	Niger	2019–6-15	MF314189	Singapore	2016–1-11
ON907580	Niger	2019–6-15	MW512441	Singapore	2015–5-4
ON908223	Niger	2019–6-15	MW512442	Singapore	2015–4-22
MT261962	Burkina Faso	2017–10–25	MW512478	Singapore	2017–1-26
MZ857220	Burkina Faso	2017–9-20	MW512439	Singapore	2015–10–17
MT261967	Burkina Faso	2017–10–9	MW512440	Singapore	2015–9-12
MT982731	Burkina Faso	2016–11–2	MW512456	Singapore	2016–4-23
MT980927	Mauritania	2017–11–30	MW512457	Singapore	2016–1-19
MT981148	Senegal	2019–1-4	MW512458	Singapore	2016–10–5
ON231310	Senegal	2018–10–31	MW512459	Singapore	2016–1-1
ON231309	Senegal	2018–10–28	MW512444	Singapore	2015–6-14
MT981011	Senegal	2018–10–15	MW512461	Singapore	2016–11–24
OK384579	Mauritania	2018–10–13	MW512443	Singapore	2015–5-30
ON231306	Senegal	2018–10–28	KU948303	Singapore	2016–2-23
ON231308	Senegal	2018–10–28	MW512460	Singapore	2016–9-21
ON231307	Senegal	2018–10–28	MW512445	Singapore	2015–12–31
GQ398263	Indonesia	1975–12–31	MW512462	Singapore	2016–1-25
GQ398264	Indonesia	1976–3-7	MK564483	China	2017–8-11
OK469352	Singapore	1993–12–31	MH827551	China	2017–1-1
KU666945	Malaysia	2014–1-31	OP895917	Maldives	2021–12–31
MW512467	Singapore	2016–1-1	OP684186	China	2018–5-28
JN851131	Singapore	2005–1-1	OP684195	China	2018–6-20
FJ196852	China	2001–10–26	MT006163	Sri Lanka	2018–7-10
KC964093	China	2001–10–26	MT006137	Sri Lanka	2017–10–23

Accession no.	Location	Collection date	Accession	Location	Collection date
KU509269	Philippines	2009–11–23	MT006146	Sri Lanka	2017–11–2
KU509275	Philippines	2008–7-15	MT006138	Sri Lanka	2017–10–25
MT832071	Philippines	2013–3-13	MT006136	Sri Lanka	2017–10–13
MT832072	Philippines	2013–3-13	MT006144	Sri Lanka	2018–4-30
MT832055	Philippines	2013–10–11	MT006157	Sri Lanka	2017–11–21
MT832056	Philippines	2013–10–11	MT006161	Sri Lanka	2017–12–13
MT832062	Philippines	2013–1-1	MT006174	Sri Lanka	2018–1-22
MW512432	Singapore	2014–12–31	MT006155	Sri Lanka	2017–11–21
MT832057	Philippines	2015–9-29	MT006170	Sri Lanka	2018–1-8
MT832058	Philippines	2015–9-29	MT006159	Sri Lanka	2017–11–24
MN944002	China	2019–7-2	MT180479	Sri Lanka	2017–8-1
MW512489	Singapore	2018–12–31	MW512477	Singapore	2017–1-1
MT832080	Philippines	2013–5-25	MN577548	Sri Lanka	2017–7-31
MT832065	Philippines	2014–1-11	MN577546	Sri Lanka	2017–8-9
MT832066	Philippines	2014–7-14	MN577549	Sri Lanka	2017–8-9
MT832053	Philippines	2014–7-23	MH827548	China	2017–4-23
MT832054	Philippines	2014–7-23	MT006175	Sri Lanka	2018–1-30
MK783205	China	2018–12–31	OM978278	Sri Lanka	2020–2-5
MT832064	Philippines	2013–12–13	OQ102952	Sri Lanka	2022–5-9
MT832077	Philippines	2013–3-17	OQ102945	Sri Lanka	2021–11–19
MW881533	China	2021–2-2	OQ102949	Sri Lanka	2022–2-15
MT832059	Philippines	2014–12–31	OQ102944	Sri Lanka	2021–11–23
MT832069	Philippines	2013–1-1	OQ102946	Sri Lanka	2021–12–1
MH827542	China	2017–1-1	MT006139	Sri Lanka	2017–10–31
MN018350	China	2016–6-29	MT006140	Sri Lanka	2017–10–31
MW512433	Singapore	2014–1-1	MT006153	Sri Lanka	2017–11–12
MH827545	China	2017–1-1	MT006173	Sri Lanka	2018–1-22
MN018364	China	2016–8-12	MT006180	Sri Lanka	2018–3-28
OM368352	China	2018–5-17	MT006142	Sri Lanka	2018–4-22
ON875316	China	2018–5-17	MT006176	Sri Lanka	2018–2-14
ON908222	China	2018–5-17	MT006186	Sri Lanka	2018–4-9
MN566109	New Caledonia	2017–4-1	MT006171	Sri Lanka	2018–1-19
MN566110	New Caledonia	2017–6-18	MT006181	Sri Lanka	2018–3-29
MH985859	Australia	2017–1-1	MT006179	Sri Lanka	2018–3-26
MN566111	New Caledonia	2018–4-9	MT006165	Sri Lanka	2018–7-18
MN566112	New Caledonia	2018–4-23	MT006150	Sri Lanka	2017–11–16
KF744398	Philippines	2005–12–31	MT006178	Sri Lanka	2018–3-15
MG599599	Taiwan	2001–5-10	MT006160	Sri Lanka	2017–12–1
MG599594	Taiwan	2001–6-22	MT006185	Sri Lanka	2018–4-3
MG599596	Taiwan	2001–6-22	MT006145	Sri Lanka	2018–4-30
DQ645542	Taiwan	2001–11–12	MT006184	Sri Lanka	2018–4-1
MG599595	Taiwan	2001–11–11	MT006167	Sri Lanka	2017–12–21
DQ645540	Taiwan	2001–10–31	MT006168	Sri Lanka	2018–1-3
MG599592	Taiwan	2001–11–1	MT006172	Sri Lanka	2018–1-19
DQ645543	Taiwan	2001–12–3	MT006164	Sri Lanka	2018–7-17
MG599597	Taiwan	2001–12–2	MT006177	Sri Lanka	2018–3-14
DQ645544	Taiwan	2001–12–7	MT006143	Sri Lanka	2018–4-25
MG599598	Taiwan	2001–12–6	MT006149	Sri Lanka	2018–5-17
DQ645541	Taiwan	2001–11–3	MW512471	Singapore	2017–1-1
MG599593	Taiwan	2001–7-3	MH827550	China	2017–12–31
MG599600	Taiwan	2001–8-11	MN018354	China	2017–5-23
MG599601	Taiwan	2001–11–13	MW512419	Singapore	2014–1-1
MG599606	Taiwan	2001–12–24	MW512472	Singapore	2017–1-1
MG599610	Taiwan	2002–1-10	MW512454	Singapore	2016–1-1
MG599617	Taiwan	2002–1-7	MW512473	Singapore	2017–1-1
MG599603	Taiwan	2002–1-7	MK564480	China	2016–8-26
DQ645555	Taiwan	2002–11–1	MZ636803	Thailand	2019–11–28
MG599630	Taiwan	2002–10–31	MZ636805	Thailand	2019–12–30
MG599602	Taiwan	2002–1-21	MN923121	China	2019–10–10
MG599609	Taiwan	2002–1-21	MH827546	China	2017–2-23
MG599633	Taiwan	2002–8-26	MN018344	China	2017–3-11
MG599608	Taiwan	2002–1-21	MH110599	China	2017–9-1
MG599612	Taiwan	2002–2-13	OP684147	China	2017–9-19
MG599611	Taiwan	2002–2-13	OP684170	China	2017–9-5
MG599613	Taiwan	2002–2-13	OP684176	China	2017–9-5
DQ645551	Taiwan	2002–9-12	OP684163	China	2017–8-29
MG599628	Taiwan	2002–12–31	MW512492	Singapore	2019–4-10
MG599619	Taiwan	2002–5-15	MN923118	China	2019–8-14
MG599607	Taiwan	2002–4-2	MW720955	China	2019–9-1

Accession no.	Location	Collection date	Accession	Location	Collection date
DQ645548	Taiwan	2002–7-20	OP684181	China	2018–7-3
DQ645545	Taiwan	2002–6-17	OQ653841	China	2022–7-22
MG599605	Taiwan	2002–6-4	OL435143	Cambodia	2020–8-1
MG599629	Taiwan	2002–7-29	MW295818	China	2020–10–9
DQ645552	Taiwan	2002–9-13	MW345921	China	2020–10–18
MG599615	Taiwan	2002–3-27	OL414747	Cambodia	2019–8-1
DQ645553	Taiwan	2002–10–23	ON887284	Cambodia	2019–7-8
MG599622	Taiwan	2002–7-14	OL412740	Cambodia	2019–7-1
MG599634	Taiwan	2002–10–10	OL414727	Cambodia	2019–8-31
DQ645546	Taiwan	2002–6-24	OL414730	Cambodia	2019–7-1
MG599618	Taiwan	2002–6-12	OL414742	Cambodia	2019–6-1
MG599626	Taiwan	2002–12–31	OL414740	Cambodia	2019–6-1
MG599620	Taiwan	2002–8-17	OL414738	Cambodia	2019–6-1
DQ645549	Taiwan	2002–8-18	OL414763	Cambodia	2019–8-1
MG599623	Taiwan	2002–3-26	OL414759	Cambodia	2019–6-30
MG599632	Taiwan	2002–11–17	OL414722	Cambodia	2019–8-1
DQ645556	Taiwan	2002–11–18	OL414725	Cambodia	2019–8-31
DQ645550	Taiwan	2002–8-19	OL414724	Cambodia	2019–8-1
MG599616	Taiwan	2002–7-17	OL414717	Cambodia	2019–7-31
DQ645547	Taiwan	2002–7-16	OL414718	Cambodia	2019–7-26
MT921572	Australia	2000–1-1	OL414760	Cambodia	2019–8-27
FJ196853	China	2003–1-1	MW295816	China	2020–8-7
FJ196854	China	1993–12–23	OL414736	Cambodia	2020–6-17
KC964094	China	1993–12–23	OL414746	Cambodia	2020–6-16
JN851124	Singapore	2005–3-6	OQ028217	Vietnam	2019–6-7
EU081179	Singapore	2005–9-5	OQ028216	Vietnam	2022–5-25
EU081178	Singapore	2005–5-19	OQ028225	Vietnam	2020–9-23
EU081177	Singapore	2005–10–22	OL414764	Cambodia	2020–8-1
JN851125	Singapore	2005–3-31	OQ678101	Cambodia	2020–6-3
JN851130	Singapore	2005–8-16	OQ683881	Cambodia	2020–6-2
KC762674	Indonesia	2008–3-11	OL414754	Cambodia	2020–8-1
KC762677	Indonesia	2008–2-15	OQ678102	Cambodia	2020–7-30
KC762675	Indonesia	2008–3-8	OL414749	Cambodia	2020–6-5
KU509270		2012–12–31	OL414732	Cambodia	2020–8-31
MW512385	Singapore	2013–1-1	OL414758	Cambodia	2020–9-12
MF004385	France	2014–9-6	OQ000263	Cambodia	2020–9-12
MW512384	Singapore	2013–1-1	OL414733	Cambodia	2020–8-1
MK564477	China	2016–4-15	OL414756	Cambodia	2020–8-31
MW512479	Singapore	2018–12–31	OL414731	Cambodia	2020–9-12
MW512450	Singapore	2016–4-23	OP999336	Cambodia	2020–9-12
OP410992	Singapore	2016–10–1	OL414753	Cambodia	2020–8-1
MH827527	China	2017–1-1	OL414765	Cambodia	2020–8-1
MN018352	China	2015–9-7	OL414752	Cambodia	2020–7-29
KU365901	Taiwan	2015–1-1	OL414755	Cambodia	2020–8-25
KU365902	Taiwan	2015–3-18	OL414757	Cambodia	2020–9-11
KU365903	Taiwan	2015–5-14	OP999339	Cambodia	2020–9-11
MW512480	Singapore	2018–12–31	OL414751	Cambodia	2020–7-14
MW945435	Vietnam	2006–12–31	OL414750	Cambodia	2020–7-14
MH048673	Malaysia	2014–12–6	OP684196	China	2019–1-5
MH048675	Malaysia	2014–12–7	OP684151	China	2017–9-26
MF043956	China	2016–1-1	MH110564	China	2017–9-13
MW721475	China	2010–10–9	MH110572	China	2017–9-13
MW721474	China	2010–10–9	MW512482	Singapore	2018–1-1
JX470186	China	2010–12–31	MW512494	Singapore	2019–1-1
KP723479	China	2010–11–21	MW512481	Singapore	2018–4-5
KC762679	Indonesia	2010–3-29	OM639979	India	2021–10–19
KC762671	Indonesia	2008–2-19	MK564485	China	2018–8-28
KC762672	Indonesia	2008–4-9	MK783202	China	2018–11–27
ON907579	China	2017–8-7	MK783203	China	2018–11–27
MK564487	China	2018–9-3	MW512483	Singapore	2018–1-1
MK783204	China	2018–8-23	MN923110	China	2019–8-4
MW721459	China	2018–10–1	MW720947	China	2019–9-1
ON887640	China	2018–8-28	MN923108	China	2019–6-20
ON908226	China	2018–8-28	ON908244	China	2019–6-20
MG189962	Tanzania	2014–6-1	MW720949	China	2019–9-1
MZ857213	Tanzania	2014–6-30	MW720946	China	2019–9-20
MZ857212	Tanzania	2014–1-1	MW720945	China	2019–9-1
MZ857214	Tanzania	2014–12–12	MN923119	China	2019–8-21
MZ857208	Kenya	2013–12–30	MW720950	China	2019–9-1

Accession no.	Location	Collection date	Accession	Location	Collection date
MZ857215	Somalia	2015–1-1	MW720957	China	2019–9-17
MZ857211	Mozambique	2014–9-9	MW720951	China	2019–9-11
MZ857210	Tanzania	2014–9-9	MW720954	China	2019–9-11
MK564478	China	2016–4-22	MW512476	Singapore	2017–1-1
KT187555	China	2014–1-7	MW512474	Singapore	2017–1-1
KT187553	China	2014–7-15	MW512488	Singapore	2018–6-2
KP012546	China	2014–12–31	MW512497	Singapore	2019–1-1
MW721465	China	2014–10–1	MW512486	Singapore	2018–10–2
KT187557	China	2014–12–23	MW512498	Singapore	2019–1-1
KP723478	China	2014–12–31	MW512487	Singapore	2018–12–31
MW721473	China	2014–10–1	MH827554	China	2017–6-13
KT187556	China	2014–5-2	MN018360	China	2017–7-2
KT187558	China	2014–7-24	MK564481	China	2017–7-27
MW721468	China	2014–10–18	MW512484	Singapore	2018–1-1
KX225486	China	2015–1-1	MW512485	Singapore	2018–8-8
MW721463	China	2014–10–28	MW186239	Singapore	2019–10–1
KX655787	China	2015–1-1	OP410990	Singapore	2019–10–31
MW721464	China	2014–10–29	MK543448	China	2018–9-23
KT187554	China	2014–11–3	MK543479	China	2018–9-23
MW721466	China	2014–10–31	MK543471	China	2018–9-23
MW721467	China	2014–10–1	MK543449	China	2018–9-26
KX655786	China	2015–1-1	OP811980	Pakistan	2022–4-28
MW721462	China	2014–10–31	OP811983	Pakistan	2022–9-28
JN851128	Singapore	2006–3-2	OP811981	Pakistan	2022–10–18
KC762658	Indonesia	2007–8-14	OP811982	Pakistan	2022–9-13
MT921573	Australia	2004–1-1	OP898559	Pakistan	2022–10–1
KC762665	Indonesia	2007–7-6	OP811984	Pakistan	2022–9-30
KC762678	Indonesia	2010–3-4	OP811978	Pakistan	2022–4-27
KC762664	Indonesia	2008–3-28	OP811977	Pakistan	2022–4-27
MW512414	Singapore	2014–12–31	OP811979	Pakistan	2022–4-28
KY794785	Papua New Guinea	2010–5-11	MW512475	Singapore	2017–1-1
MT921570	Australia	2013–1-1	MH110565	China	2017–9-1
KU517845	Papua New Guinea	2013–4-23	MH110566	China	2017–9-1
OL321183	Papua New Guinea	2016–3-12	MH110576	China	2017–8-1
OL321186	Papua New Guinea	2016–4-12	MH110597	China	2017–9-1
OL321176	Papua New Guinea	2016–1-1	OP684156	China	2017–8-22
MH985858	Australia	2016–1-1	OP684172	China	2017–9-6
OP684198	China	2019–1-20	OP684144	China	2017–9-18
OL321182	Papua New Guinea	2016–3-5	OP684148	China	2017–9-21
OL321184	Papua New Guinea	2016–5-10	OP684179	China	2017–9-12
OL321178	Papua New Guinea	2016–3-7	OP684146	China	2017–9-23
OL321185	Papua New Guinea	2016–4-21	OP684178	China	2017–9-11
MT921571	Papua New Guinea	2015–12–16	OP684145	China	2017–9-20
MN018363	China	2016–2-23	OP684149	China	2017–9-26
OL321177	Papua New Guinea	2016–3-28	OP684175	China	2017–9-5
KC762657	Indonesia	2008–4-23	OP684177	China	2017–9-7
KC762659	Indonesia	2008–4-15	OP684171	China	2017–8-24
KX452015	Malaysia	2014–1-31	MH110594	China	2017–8-1
KM279604	Singapore	2008–4-19	MH110598	China	2017–9-1
MW512356	Singapore	2009–7-18	MH110579	China	2017–8-17
KU509272	Thailand	2009–12–31	MH110580	China	2017–8-31
KM279605	Singapore	2009–1-1	OP684165	China	2017–9-1
MW512361	Singapore	2010–12–31	OP684168	China	2017–9-3
KM279606	Singapore	2010–7-6	OP684158	China	2017–8-29
MW512359	Singapore	2010–12–3	OP684164	China	2017–8-29
JN851113	Singapore	2006–11–7	OP684157	China	2017–8-30
OP684167	China	2017–9-2	MH110590	China	2017–9-30
